# Substrate stiffness-dependent metabolic reprogramming of iPSC-derived cardiomyocytes on physiological PDMS polymers

**DOI:** 10.1016/j.mec.2025.e00266

**Published:** 2025-07-08

**Authors:** Leena Patel, Bryan P. Marzullo, Jonathan Barlow, Himani Rana, Amar J. Azad, Patricia Thomas, Daniel A. Tennant, Katja Gehmlich

**Affiliations:** a Department of Cardiovascular Sciences, School of Medical Sciences, University of Birmingham, Birmingham, UK; b Department of Metabolism and Systems Science, School of Medical Sciences, University of Birmingham, Birmingham, UK; c Cellular Health and Metabolism Facility, School of Sport, Exercise and Rehabilitation Sciences, University of Birmingham, Birmingham, UK; d Division of Cardiovascular Medicine, Radcliffe Department of Medicine and British Heart Foundation Centre of Research Excellence Oxford, University of Oxford, Oxford, UK

## Abstract

Many cardiac pathologies are characterised by increased stiffness of the myocardium, due to excess deposition of extracellular matrix (ECM) proteins and structural remodelling, impacting the behaviour of cardiomyocytes (CMs). Metabolism of CMs shifts in cardiac pathologies, with the healthy heart primarily utilising fatty acids as its source of energy production, whilst the diseased heart switches to utilise glucose. The shift in metabolic source with stiffness of the myocardium has not been investigated.

To investigate the effect of ECM stiffnesses on iPSC-CM metabolism, iPSC-CMs were cultured on polydimethylsiloxane (PDMS) substrates of healthy and fibrotic stiffness (20 kPa and 130 kPa respectively) and plastic. Cellular metabolism of iPSC-CMs was assessed through isotope-labelled mass spectrometry with central carbon tracing as well as real-time cellular bioenergetics using extracellular flux analysis. Key metabolic genes were investigated at transcript and protein level, with proteomics analysis conducted to identify protein profiles on substrate stiffnesses.

Mass spectrometry data revealed greater utilisation of glucose in iPSC-CMs cultured on plastic compared to softer PDMS substrates, indicating greater glycolytic activity. Extracellular flux analysis demonstrated greater lactic acid efflux from iPSC-CMs cultured on plastic substrates, reflective of increased glycolytic flux and a shift towards aerobic glycolysis as the primary source of ATP synthesis. This study revealed culture of iPSC-CMs on traditional cell culture plastics or glass coverslips displaying pathological metabolism, highlighting the use of physiological substrates for metabolic investigation.

## Highlights

Patel et al. Highlights:

- Glass/plastic routinely used to culture cardiomyocytes is non-physiologically stiff
- Viscoelastic PDMS polymers provide softer, fine tuneable substrates for cells
- Stem cell derived cardiomyocytes can be cultured on PDMS substrates
- Softer PDMS substrates shift the metabolism of these cells away from glycolysis
- Glass/plastic stiff substrates are an artificial condition with changed metabolism

## 1 Introduction

Cardiovascular disease (CVD) is a leading cause of mortality worldwide, with heart and circulatory diseases responsible for approximately 26 % of total deaths in the UK (British Heart Foundation, 2025). Epidemiological evidence has demonstrated increased incidences of CVD with age, from 40 % between ages of 40–59, to 75 % between the ages of 60–79 and 86 % in those aged above 80 years (Yazdanyar and Newman, 2009). Cardiac pathologies have been associated with a decline in physiological processes, with the heart undergoing a series of molecular and cellular changes, such as enhanced ECM deposition and fibrosis (Burkauskiene, 2005; Bradshaw et al., 2010; Annoni et al., 1998). Such increased deposition of collagen and ECM proteins causes increased stiffness of the ECM, impairing function of cardiac cells and often leading to contractile dysfunction (Travers et al., 2016; Jiang et al., 2021). The increased stiffness and rigidity of the ECM can consequently impact CM behaviour, altering phenotypes and structure (Cho et al., 2018).

**Fig. 1:**
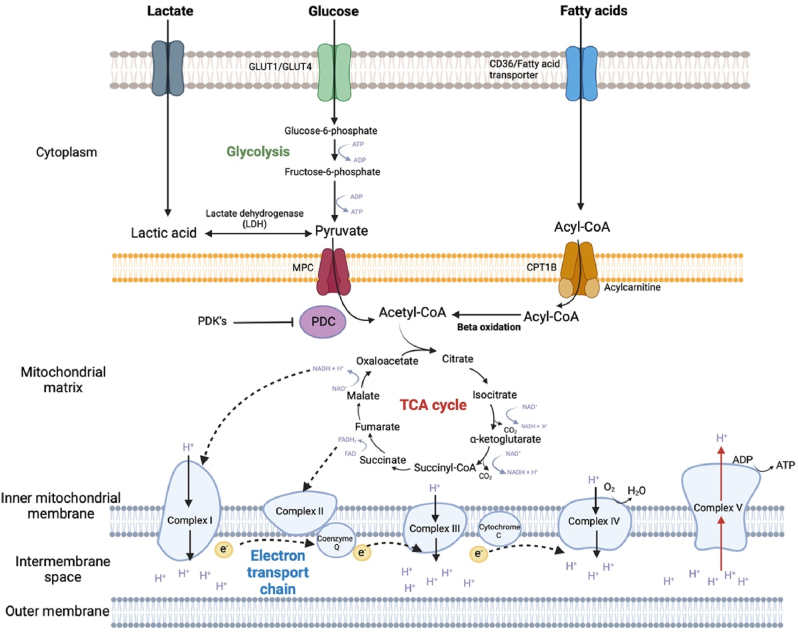
**Metabolic substrates and pathways used for energy production in cardiomyocytes** Metabolism of glucose, lactate and fatty acids. Glucose is broken down into pyruvate via Glycolysis. Pyruvate is consequently transported into the mitochondria using the mitochondrial pyruvate carrier, and is oxidised into acetyl-CoA by the pyruvate dehydrogenase complex (PDC). Acetyl-CoA enters the citric acid cycle (TCA) where it forms reducing equivalents, NADH and FADH2. These electrons are transported to the electron transport chain (ETC) where they are transferred along protein complexes to molecular oxygen (as the final electron acceptor) at complex IV. This in turn drives proton extrusion from the mitochondrial matrix at complex I, III and IV creating an electro-chemical gradient. As protons return through ATP synthetase (Complex V), ADP is phosphorylated to ATP. Adapted from (Ng et al., 2023).

Induced pluripotent stem cell derived CMs (iPSC-CMs) have emerged as a novel system to study cardiac physiology and pathophysiology. Even though they more resemble foetal like cardiomyocytes, they can be pushed to adapt more adult like characteristics – a process called maturation – and iPSC-CMs are now widely used in the cardiovascular field. Structural changes and maturation of iPSC-CMs can alter the metabolic function of mitochondria and cells, which are particularly important for CM contractility and maintaining high energetic demand (Garbern and Lee, 2021). Under physiological conditions, a healthy adult heart obtains 95 % of its ATP from oxidative phosphorylation of fatty acids, with the remaining 5 % coming from glycolysis and the citric acid cycle (TCA) (Lopaschuk et al., 2010) (Fig. 1). However, in pathological settings, the availability of energy substrates shifts, with a switch towards increased glycolysis at the expense of lipid metabolism (Fukushima et al., 2015). E.g. failing hearts exhibit a 30–40 % reduction in ATP synthesis (Karwi et al., 2018). The shift to glycolysis reflects a switch back to foetal like metabolism (Morciano et al., 2021), and often occurs in cardiac diseases such as heart failure and cardiac hypertrophy (Tran and Wang, 2019).

To investigate the effect of ECM stiffness on CM function, representative models of physiological stiffnesses of the ECM are required. Current experiments conducted on cell culture plastics and glass hold stiffnesses of 1–70 GPa, which are far stiffer than the healthy myocardium, which has stiffnesses of approximately 10–20 kPa (Ahmed et al., 2020; Felisbino et al., 2024). Viscoelastic polymers are three-dimensional (3D) crosslinked networks formed from biomaterials, which can be mechanically modified to alter stiffnesses by varying crosslinking density of polymer chains, with increased crosslinking leading to increased stiffness (Drury and Mooney, 2003; Catoira et al., 2019). Synthetic biomaterials such as Polydimethylsiloxane (PDMS) have been used in the cardiac field widely, due to low cost, optical transparency of gels, ease of fabrication and delivery for bioactive molecules (Lin and Anseth, 2009; Martin and Bhushan, 2017). Varying models of PDMS viscoelastic polymers have been utilised for studying cardiac diseases such as fibrosis, as cardiac fibroblasts cultured on stiffer PDMS viscoelastic polymers portrayed activation and conversion to myofibroblasts (Yazdanyar and Newman, 2009), indicating that PDMS can mirror pathophysiological conditions of the ECM.

There is little evidence to portray whether there is a link between stiffening of the myocardium and the shift in cardiac metabolism occurring in pathological settings, highlighting the importance of investigating the potential link between the two. As CMs play a significant role in cardiac metabolism, modelling physiological conditions of the ECM and investigating changes in iPSC-CMs on these substrates can help elucidate molecular mechanisms through which stiffness may alter metabolic regulation. No previously published studies have assessed changes in central carbon metabolism in iPSC-CMs cultured on PDMS substrates representative of physiological stiffnesses.

The main objective of this research was to investigate whether changes in the stiffness of the ECM, modelled by iPSC-CMs cultured on PDMS viscoelastic polymers of 20 kPa PDMS, 130 kPa PDMS and plastic (1–25 GPa), cause a shift in metabolic substrate utilisation. This would be reflective of the stiffer environment cardiomyocytes experience in cardiac diseases.

## 2 Methods

### 2.1 PDMS viscoelastic polymer synthesis

PDMS substrate viscoelastic polymers were prepared using Sylgard 527 (Dow, cat no.1675167) and Sylgard 184 (Dow, 63416-5S). PDMS stiffnesses of 20 kPa and 130 kPa were produced by adding different mass ratios of Sylgard 184: Sylgard 527, 1:10 and 1:5 respectively. PDMS gels in plates were cured overnight at 65 °C before undergoing phosphate buffered solution (PBS) washes, followed by 70 % ethanol sterilisation for 1 h at room temperature before further PBS washes to remove any remaining ethanol or unreacted precursors. PDMS gels were coated with Geltrex at 1:100 (ThermoFisher Scientific, A1413202) before iPSC-CMs were plated onto the gels. Stiffnesses of PDMS viscoelastic polymers were tested using rheological characterisation (Supplementary Fig. 2) and Young's modulus stiffnesses calculated using the following formulas:

$$E=2G\;\left(1+v\right)\;\text{and}\;G={\surd G}^{\prime }2+\surd G^{\prime \prime }2$$

### 2.2 Differentiation of ventricular induced pluripotent stem cell-derived cardiomyocytes

iPSC cells were differentiated into ventricular cardiomyocytes using an established and published protocol (Cumberland et al., 2023) (Supplementary Fig. 1) on standard tissue culture plastic. Briefly, iPSCs were plated and cultured with initiation of differentiation at 70–80 % confluency. Beating CMs were established on days 8–10, with iPSC-CMs passaged onto PDMS viscoelastic polymers of 20 kPa, 130 kPa and plastic wells between days 12–15 of differentiation. iPSC-CMs were cultured on PDMS viscoelastic polymers until the day of experiment, typically day 25. Control cells were kept on standard tissue culture plastic, or glass coverslips for immunofluorescence.

### 2.3 Isotope label Gas Chromatography Mass Spectrometry

iPSC-CMs were differentiated and seeded onto PDMS viscoelastic polymers between days 12–15 at approximately 50,000 cells per cm^2^ for Gas Chromatography Mass Spectrometry (GC-MS). Oleic acid (OA) and palmitic acid (PA) were conjugated to bovine serum albumin (BSA). Briefly, OA and PA were dissolved in 70 % ethanol at 70 °C to make 10 mM stocks. 10 % BSA in RPMI 1640 media with a B27 + Insulin supplement (RPMI + Ins, Gibco) solution, 11 mM glucose was also made at 37 °C (BSA media). OA and PA stocks were heated to 70 °C and added at a 1:10 ratio to warmed BSA media, and left to warm in a 37 °C water bath for 1 h. A further 1:10 dilution of the fatty acids in BSA media was added to RPMI + Ins media, filtered and added to the iPSC-CMs. Cells were supplemented with total fatty acid concentrations of 50 μM PA and 100 μM OA in RPMI + Ins media for a period of 10 days, with media removed and replaced every other day. 10 mM isotopically labelled D-Glucose (U-^13^C-glucose, 99 % Cambridge Isotope Laboratories) was added to the SILAC RPMI 1640 Flex media supplemented with 1.14 mM L-Arginine, 0.2 mM L-lysine, 2 mM L-Glutamine and B27+Ins supplement. SILAC labelling media was also made up with unlabelled 10 mM D-Glucose for control samples. Day 26 iPSC-CMs were incubated with SILAC labelling media with or without labelled glucose for 48 h. Polar metabolite extraction was conducted on ice. Briefly, iPSC-CMs were quenched with 500 μl of ice cold HPLC grade methanol. 200 μl of GC-MS grade water containing 1 μg/ml pentanedioic-d6 acid as internal standard (C/D/N Isotopes Inc, D-5227) was added to the methanol on iPSC-CMs and scraped using a cell scraper. Solutions were vortexed to ensure cell lysis. 500 μl of HPLC grade chloroform was added to samples and vortexed, producing a cloudy mixture. Samples were centrifuged at 14,800g for 10 min at 4 °C. 400 μl of the upper phase solution containing the polar metabolites were transferred to a new Eppendorf tube. Samples were dried using a SpeedVac vacuum centrifuge at 37 °C (ThermoFisher Scientific) for 2–3 h and stored at −80 °C until derivatisation. 20 μl of 2 % methoxyamine hydrochloride and pyridine (Sigma-Aldrich) was added to the dried samples and placed on a shaker for 10 min at room temperature. Following shaking, samples were incubated at 60 °C for 1 h on a heating block. 30 μl of N-methyl-N[tertbutyldimethylsilyltrifluoroacetamide] (MTBSFA) with 1 % tert-butyldimethyl-chlorosilane (TBDMSC) (Restek) were added to the samples, shaken for 10 min at room temperature and heated for 1 h at 60 °C on a heating block. Samples were centrifuged for 15 min at 3000g and solution was transferred into GC-MS vials with glass inserts. Samples were run on an Agilent 7890B GC and 5977A MSD. 1 μl of sample was injected in splitless mode with helium carrier gas at a rate of 1 ml/min, sample inlet was 270 °C. Initial oven temperature was held at 100 °C for 1 min before ramping to 160 °C at a rate of 10 °C/min, followed by a ramp to 200 °C at a rate of 5 °C/min, and a final ramp to 330 °C at a rate of 10 °C/min with a 4 min hold. Transfer line was held at 270 °C. Analytes were ionised via EI (70 eV) and source temperature of 230 °C. Compound detection was carried out in scan mode.

### 2.4 Real-time cellular metabolic profiling

XF^e^96 well microplates (Agilent Technologies) were pre-coated with 5 μl of 20 kPa PDMS viscoelastic polymer, 5 μl 130 kPa PDMS viscoelastic polymer or kept as plastic. PDMS coated plates were washed with PBS, followed by 70 % ethanol for 1 h and further washed in PBS before coating with Geltrex (add details of product or include final concentration) at 1:100. iPSC-CMs were then seeded into pre-coated XF^e^96 cell culture plates at approximately 20,000 cells per well. Wells A1, H1, A12 and H12 were kept empty and used as background correction wells. Real-time cellular metabolic profiling was assessed as previously described (Patel et al., 2024). In brief, following 4 baseline measures consisting of a 3-min wait and 3-min measure cycle, the sequential addition of Oligomycin (2 μg/ml), BAM15 (3 μM), Rotenone plus Antimycin A (2 μM) and Monensin (20 μM) were added to establish basal energetic activity, ATP-coupled respiration, mitochondrial proton leak, maximal mitochondrial respiratory capacity, spare mitochondrial respiratory capacity, compensatory glycolysis and glycolytic capacity, using oxygen consumption rate (OCR) and proton efflux rate (PER) data. Three measurements, were obtained after each drug addition. Post XF analysis, cell plates were centrifuged at 220×*g* for 5 min to pellet iPSC-CMs prior to media removal. Cell pellets were lysed in 10 μl RIPA buffer and protein concentrations determined using a Pierce Rapid Gold BCA Protein Assay Kit. Data were analysed using Agilent Seahorse analytics (version 1.0.0–699) and MS Excel for Mac.

### 2.5 Proteomics

Day 25 iPSC-CMs cultured on PDMS substrates and plastic were pelleted and processed using the EasyPep Mini MS Sample Prep Kit (ThermoFisher Scientific, A40006) as per stated in the protocol. 40 μg of protein from each sample was used for reduction and alkylation. Samples were digested overnight in a shaking incubator at 37 °C at 150 rpm. Peptides were labelled with TMTpro™ 16plex Mass Tag Labelling Reagent Set (ThermoFisher Scientific, A44522). Clean-up of peptides using columns from the EasyPep Mini MS Sample Prep Kit was conducted until samples were in elution solution. The samples were taken to the Advanced Mass Spectrometry Facility at University of Birmingham, with mass spectrometry and quantitative proteomics runs conducted by Todd Mize. Samples were combined into one sample and underwent a ZipTip clean up (Merck Millipore, ZTC18S096). Samples were run on Precolumn Cartridges, Acclaim PepMap 100 C18, 5 μm 100A,300 μmi.d.x5mm(Dionex) and separated in a Nano Series TM Standard Columns 75 μm i.d. x 15 cm, packed with C18 PepMap100, 3 μm, 100A (Dionex). Gradients of 3.2 %–44 % of Solvent B (0.1 % formic acid in acetonitrile) were used for 30 min. Columns were then washed with 90 % solvent B solution and further equilibrated with 3.2 % solvent B. Peptides were eluted at 350 nL/min-1 via a Triversa Nanomate nanospray source (Advion Biosciences) into a QExactive HF Orbitrap mass spectrometer (ThermoFisher Scientific). Full FT-MS scans (m/z 375–1600) and high energy collision dissociation MS/MS scans of the most abundant ions were conducted. Mass spectra were recorded at a resolution of 120,000 at m/z 200 and autonomic gain control of 3x106. Precursor ions were fragmented in HCD MS/MS with a set resolution of 60,000 and a normalised collision energy of 32. The autonomic gain control target for HCD MS/MS was 1x105. The precursor isolation window was set at 1.2 m/z and only multiply-charged precursor ions were selected for MS/MS. Spectra were obtained for 60 min.

Statistical quantification was conducted in Proteome Discoverer (version 2.5) using ANOVA, with protein volcano plots plotted in GraphPad Prism 9. A false discovery rate threshold was set at 0.05. A threshold of significance was set as p < 0.05, log2 fold change of significance set to 0.58, equivalent to a log fold change of >1.5. The precursor mass tolerance was set at 10 ppm and a MS/MS tolerance of 0.02 Da was set for processing and quantification.

### 2.6 Real-time quantitative polymerase chain reaction (RT-qPCR)

iPSC-CMs cultured on 20 kPa PDMS, 130 kPa PDMS and plastic were collected on day 25 of differentiation. TrypLE express enzyme (ThermoFisher Scientific) was used to dissociate cells, spun down at 215g for 3 min and collected in an Eppendorf tube. iPSC-CMs were resuspended in PBS and spun down at 13000g at 4 °C for 5 min, followed by aspiration of PBS. RNA was extracted from cell pellets cultured using an RNEasy Mini Kit (Qiagen, 74104) and QIAshredder homogenizers (Qiagen,79656), as per the Qiagen Mini protocol. RNA from iPSC-CMs were converted to cDNA using Reverse transcriptase PCR, as per the High-Capacity cDNA Reverse Transcription Kit protocol (Applied Biosystems, 4368814). Master mixes contained reverse transcriptase buffer, deoxyribonucleotide triphosphate (dNTPs), reverse transcriptase primers, reverse transcriptase, nuclease-free water and 200 ng RNA in a 20 μl volume reaction. cDNA conversion was carried out using a MiniAmp Plus thermal cycler (Applied Biosystems), under the following conditions: 25 °C for 10 min, 37 °C for 120 min, 85 °C for 5 min, 4 °C hold. cDNA samples were stored in −80 °C until use.

RT-qPCR was conducted to assess transcriptional levels of metabolic genes in iPSC-CMs cultured on 20 kPa PDMS, 130 kPa PDMS and plastic. TaqMan probes (Supplementary Table 1) were used with TaqMan Fast Advanced Master Mix 2 × (Applied Biosystems, 4444557). qPCR assays were conducted on the QuantStudio™ 5 Real-Time PCR System (Applied Biosystems). The following genes were investigated: *PPARδ* (Peroxisome proliferator activated receptor delta), *PPARα* (Peroxisome proliferator activated receptor alpha), *PPARγ* (Peroxisome proliferator activated receptor gamma), *PDK4* (Pyruvate dehydrogenase kinase 4), *CD36* (Fatty acid transporter), *CPT1B* (Carnitine palmitoyltransferase 1B), *HK2* (Hexokinase 2) and *PFKM* (Phosphofructokinase). Fold changes in gene transcript expression were calculated using the ΔΔCt method, with 2-Δ(ΔCt) using *TBP* as the housekeeping gene. Data was made relative to iPSC-CMs on plastic expression levels. Cardiac markers, myosin heavy chain 7 (*MYH7*), myosin heavy chain 6 (*MYH6*), myosin light chain 2 (*MYL2*) and myosin light chain 7 (*MYL7*) were also assessed. Ratios were calculated by dividing *MYH7* by *MYH6* values, and *MYL2* by *MYL7* values.

### 2.7 Western blotting

iPSC-CM pellets were collected at day 25 in the same manner as for qPCR. Pellets were resuspended in 50 μl of 2X SDS buffer (consisting of 100 mM Tris-HCL pH 6.8, 4 % sodium dodecyl sulfate, 0.2 % bromophenol blue, 200 mM dithiothreitol and 20 % glycerol) and denatured at 95 °C for 5 min. Protein samples then underwent sonication. Protein samples were run on a 4–15 % Polyacrylamide SDS-PAGE gels in a 1X Tris-Glycine-SDS buffer and transferred to nitrocellulose membranes using an iBlot 2 Gel Transfer (Invitrogen). Blot membranes were blocked using 5 % skimmed milk in 0.1 % Tween-20 in Tris buffer saline (TBST) for 1 h, before primary antibodies were added in 5 % milk in 0.1 % TBST. Primary antibodies, rabbit PPARδ (PA1-823A, Invitrogen) at 1:750, rabbit PDK4 (ProteinTech,12949-1-AP) at 1:1000 and mouse GAPDH (Cell Signalling Technology, D4C6R) at 1:1000 were diluted in 5 % milk TBST and left overnight at 4 °C on a shaker. Membranes were washed 3 times with 0.1 % TBST for approximately 15 min. Secondary antibodies, HRP-anti rabbit (Cytiva, NA934V) at 1:5000 and IR800 anti-mouse dye (Cytiva, NXA931V) at 1:5000 were then added to 1 % milk in 0.1 % TBST for 1 h at room temperature. Washes were conducted as described above. HRP antibodies were activated using SuperSignal™ West PICO Plus Chemiluminescent Substrate (ThermoFisher Scientific) and membranes were imaged for protein bands using the Odyssey Fc Imaging Machine (LI-COR), detected at 700 nm and 800 nm. Protein quantification was carried out using ImageJ software (version 2.9).

### 2.8 Immunofluorescence

Day 25 iPSC-CMs were fixed with 4 % Paraformaldehyde in PBS for 15 min at room temperature. Cells were washed in PBS for 5 min with washes repeated three times. Fixed cells were permeabilised with blocking buffer, consisting of 0.5 % Triton-X-100 (Sigma, T8787) in PBS, 5 % Fetal Bovine Serum (FBS) (Gibco, A4766801) and 1 % BSA (Sigma-Aldrich, A3311) for 1 h at room temperature. Primary antibody, rabbit α-actinin (Ab68167, Abcam) at 1:500 was diluted in blocking buffer and applied to cells overnight at 4 °C. The following day, primary antibodies were washed off three times for 5 min each using PBS. Secondary antibodies, phalloidin F-actin (A22284) at 1:500 and Alexa Flour 568 anti-rabbit IgG (A-11011) at 1:200 were diluted in blocking buffer and added to cells for 1 h at room temperature in the dark, followed by PBS washes as above. Cells on coverslips were mounted onto glass microscopy slides using Hydromount mounting medium (National Diagnostics).

Coverslips were imaged using confocal microscopy (Zeiss LSM780 and LSM880) using a C-Apochromat 63x/1.20 W Korr M27 objective oil immersion lense, a LD LCI Plan-Apochromat 25×/0.8 lmm Korr DIC M27 water lense or an alpha Plan- Apochromat 100x/1.46 Oil DIC M27 Elyra lense. Images were saved as 8-bit or 16-bit format on Zen Blue (v. 3.1) or Zen Black Software (v.3.0). Confocal microscopy Images of α-actinin and phalloidin staining were analysed for sarcomere lengths and Z-line lengths using a Z-line detection script described in (Morris et al., 2020) in MATLAB version (R2022b) 9.13.0.2049777.

### 2.9 Statistical analysis

When comparing between multiple groups, data was assessed for normality using a Shapiro-Wilk test. Data that was not normally distributed underwent a Kruskal-Wallis test with Dunn's multiple comparisons test. Data that was normally distributed underwent one-way ANOVA with šidák post hoc test. Significance was determined as p < 0.05 (∗), <0.01 (∗∗), <0.001 (∗∗∗), <0.0001 (∗∗∗∗). Data was analysed and presented using Graphpad Prism (version 9.5.1).

## 3 Results

### 3.1 Cardiac characterisation of iPSC-CMs on PDMS substrates

**Fig. 2:**
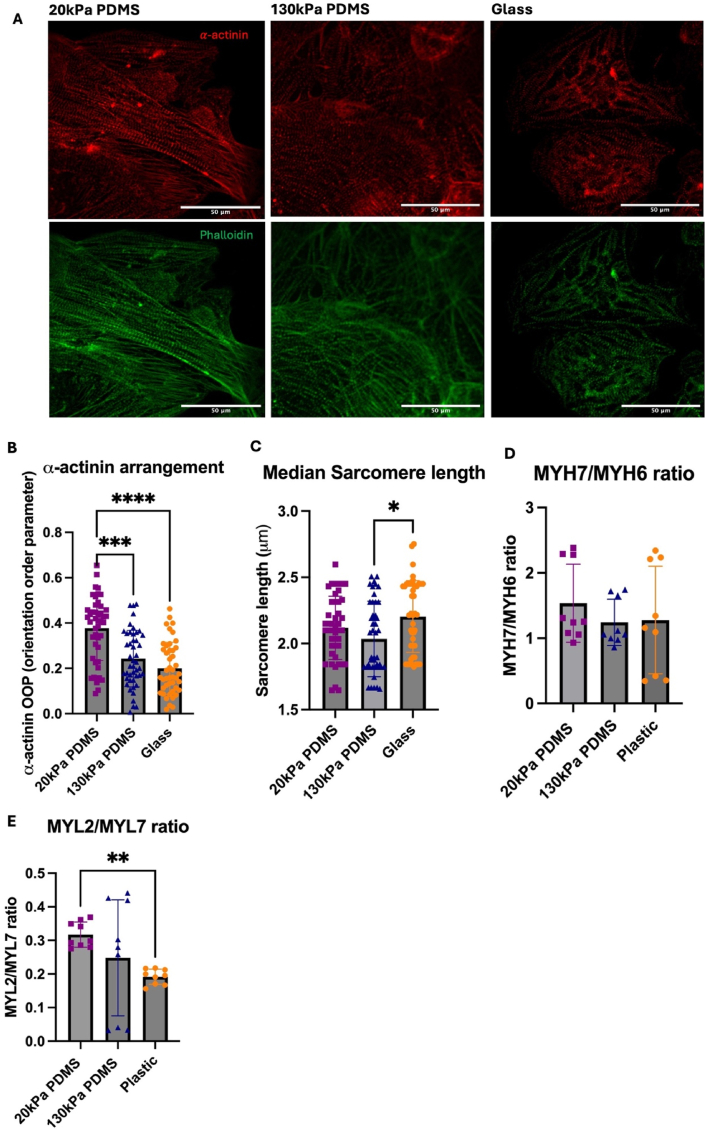
**Cardiac characterisation of iPSC-CMs cultured on PDMS substrates and plastic.** (A) Sarcomere organisation assessed by confocal microscopy images of iPSC-CMs cultured on 20 kPa PDMS, 130 kPa PDMS and glass coverslips. (B) Quantification of α-actinin organisation of iPSC-CMs across substrates. Data presented as mean ± SD, n = 4 batches/biological repeats, 1–2 wells per condition, 5–6 images per well. Each data point refers to 1 image taken from 1 well of cells across 5 technical replicates, with 2 wells per condition. A total of n = 4 batches were used (C) *MYH7*/*MYH6* ratio transcript levels of iPSC-CMs cultured on 20 kPa PDMS, 130 kPa and plastic, measured by qPCR (D) *MYL2*/*MYL7* ratio transcript levels, measured by qPCR. Each data point refers to 1 well plated as 3 technical replicates, across a total of n = 3 batches. Statistical significance was assessed with a Kruskal-Wallis test as data was not normally distributed, with p values determined as <0.01 (∗∗), <0.001 (∗∗∗), <0.0001(∗∗∗∗).

iPSC-CMs on softer substrates of 20 kPa PDMS displayed significantly greater α-actinin arrangement and sarcomere alignment compared to 130 kPa PDMS and glass, which showed disorganised sarcomere alignment (Fig. 2 A&B). Sarcomere lengths of 2.2 μm were found in iPSC-CMs cultured on glass, which were significantly longer (7.7 % increase) compared to shorter sarcomere lengths of 2.03 μm on 130 kPa PDMS (Fig. 2C). No significant differences were observed in transcript expression of *MYH7*/*MYH6* ratios in iPSC-CMs cultured on 20 kPa PDMS and 130 kPa PDMS compared to plastic surfaces (Fig. 2D). *MYL2/MYL7* transcript expression was significantly downregulated in iPSC-CMs on plastic compared to softer 20 kPa PDMS (Fig. 2E).

### 3.2 Glucose utilisation of iPSC-CMs using mass spectrometry

**Fig. 3:**
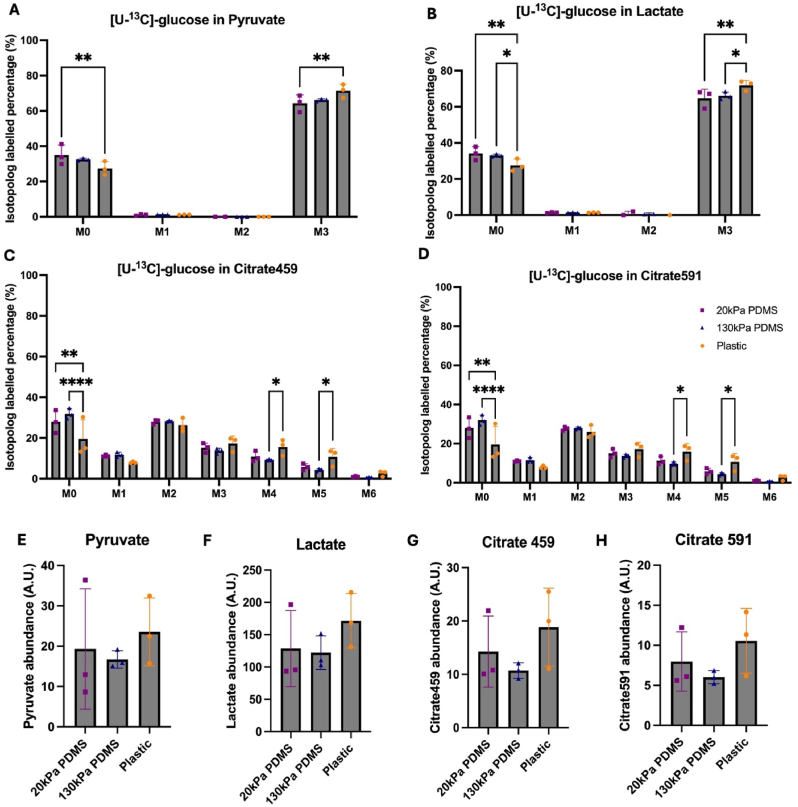
**U-**^**13**^**C-glucose labelling in metabolites of iPSC-CMs cultured on 20 kPa PDMS, 130 kPa PDMS and Plastic using isotope-labelled mass spectrometry.** Percentage of labelled U-^13^C-glucose in (A) Pyruvate (B) Lactate (C) Citrate459 (D) Citrate591. Statistical significance was assessed with a 2-way ANOVA test as data was normally distributed, with p values determined as <0.05 (∗), <0.01 (∗∗). Total abundance of (E) Pyruvate (F) Lactate (G) Citrate459 (H) Citrate591. Samples extracted from day 29 iPSC-CMs cultured on 20 kPa PDMS, 130 kPa PDMS and plastic. M0, M1, M2 and M3 refer to mass isotopomer molecules with the number of labelled carbons. Data presented as mean ± SD. Each data point refers to 1 well from 1 batch of iPSC-CMs, with a total of n = 3 batches of differentiations per condition. Data normalised to cell number from plates differentiated in parallel under same conditions. Statistical significance was assessed with a one-way ANOVA test as data was normally distributed. No significant differences were observed.

Stable isotope-enriched nutrients were incubated with iPSC-CMs and their incorporation into cellular metabolites was used to examine the metabolism of iPSC-CMs on different substrates. The percentage of U-^13^C-glucose converted into pyruvate (M+3) was significantly higher in iPSC-CMs cultured on plastic compared to 20 kPa PDMS (71 % compared to 64 %) (Fig. 3A). Similar trends were observed in the amount of U-^13^C-glucose converted into lactate (M+3); the percentage was significantly higher in iPSC-CMs on plastic compared to 20 kPa PDMS (p < 0.01) and 130 kPa PDMS (p < 0.05) (Fig. 3B). Greater amounts of unlabelled glucose (M0) was incorporated into pyruvate, lactate and citrate in iPSC-CMs cultured on 20 kPa softer substrates compared to stiffer, suggesting alternative pathways in addition to glycolysis may be altered with stiffness. The percentage of U-^13^C-glucose in acetyl-coA converted into citrate (M+2 isotopologue) showed no significant differences in iPSC-CMs across substrates (Fig. 3C). Significant differences in the proportion of glucose incorporation into citrate (M+4 and M+5 isotopologues) (Fig. 3 C&D) may reflect the increased entry of carbons through carboxylation of pyruvate alongside through acetyl-CoA. The same patterns of glucose labelling were observed in a different fragment observed for citrate (Citrate591).

Total abundance of metabolites in iPSC-CMs across substrates were also assessed (Fig. 3E–H). Despite no significant changes in the total amount of metabolites, a trend towards increased amounts of pyruvate, lactate and citrate were observed in iPSC-CMs cultured on plastic compared to softer PDMS substrates.

### 3.3 Real-time cellular metabolic profiling of iPSC-CMs

**Fig. 4:**
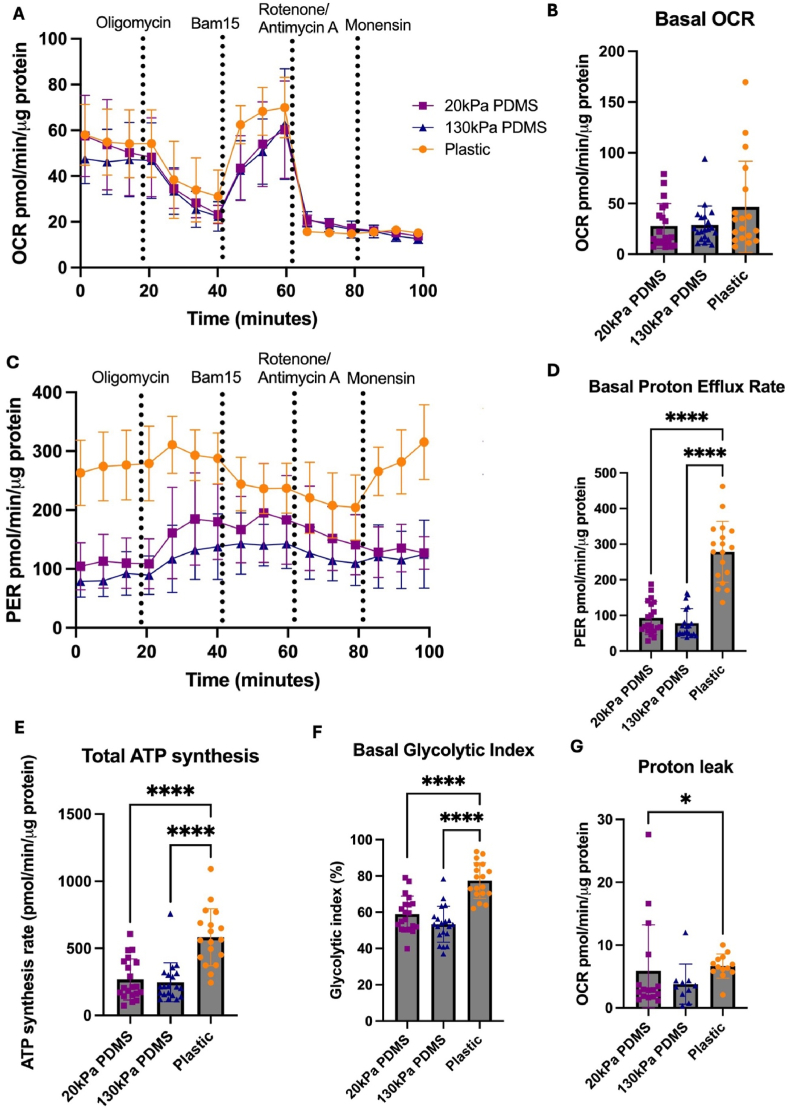
**Bioenergetic parameters assessed by real-time metabolic profiling on day 25 of iPSC-CMs cultured on 20 kPa PDMS, 130 kPa PDMS and Plastic** (A) Representative oxygen consumption rate (OCR) curves of iPSC-CMs cultured on 20 kPa PDMS, 130 kPa PDMS and Plastic. (B) Quantification of basal OCR (C) Representative proton efflux rate (PER) curves of iPSC-CMs plated on 20 kPa PDMS, 130 kPa PDMS and plastic conditions. (D) Quantification of basal PER (E) Total ATP synthesis (F) Basal glycolytic index. (G) Proton leak Data were normalised to total protein. Values are presented as mean ± SD. Each data point refers to 16 wells of cells per condition per batch, conducted using an n = 2 batches of differentiated iPSC-CMs. Statistical significance was assessed with a Kruskal-Wallis test as data was not normally distributed, with p values determined as < 0.05, 0.001 (∗∗∗), <0.0001 (∗∗∗∗). For effect sizes (Cohen's d), see Supplementary Table S2.

Basal or maximum OCR of iPSC-CMs cultured on different substrates were not significantly different (Fig. 4 A&B, Supplementary Fig. 3). Proton efflux rate (PER) refers to the number of protons exported by CMs into the media, from pyruvate catabolism to lactate and CO_2_ dissociation to bicarbonate. iPSC-CMs on plastic have a significantly higher PER compared to both 20 kPa (p < 0.001) and 130 kPa PDMS substrates (p < 0.0001), suggesting higher proton efflux on stiff substrates (Fig. 4 C&D). Since, basal mitochondrial OCR of iPSC-CMs is not significantly increased on plastic compared to viscoelastic polymers (Fig. 4B), the increase in basal PER (Fig. 4D) is likely associated with increased lactate efflux. This is consistent with an increase in total ATP synthesis (Fig. 4E) and evidenced further by a significant increase in the glycolytic index (Fig. 4F) (the proportion of glycolytic ATP as a percentage of total ATP) of iPSC-CMs cultured on plastic compared to both 20 kPa and 130 kPa PDMS (Fig. 4E and D). iPSC-CMs on plastic have significantly higher proton leak compared to those cultured on 20kPA soft PDMS substrates (Fig. 4G).

### 3.4 Proteomics analysis

Proteomics analysis was conducted to identify differentially expressed proteins in iPSC-CMs cultured on different stiffnesses. 857 proteins were identified in total before filtering. Differentially expressed proteins were identified when the fold change threshold was set to >1.5, with very few proteins reaching both the fold-change criteria and statistical significance adjusted p value threshold (<0.05, Supplementary Fig. 4).

**Fig. 5:**
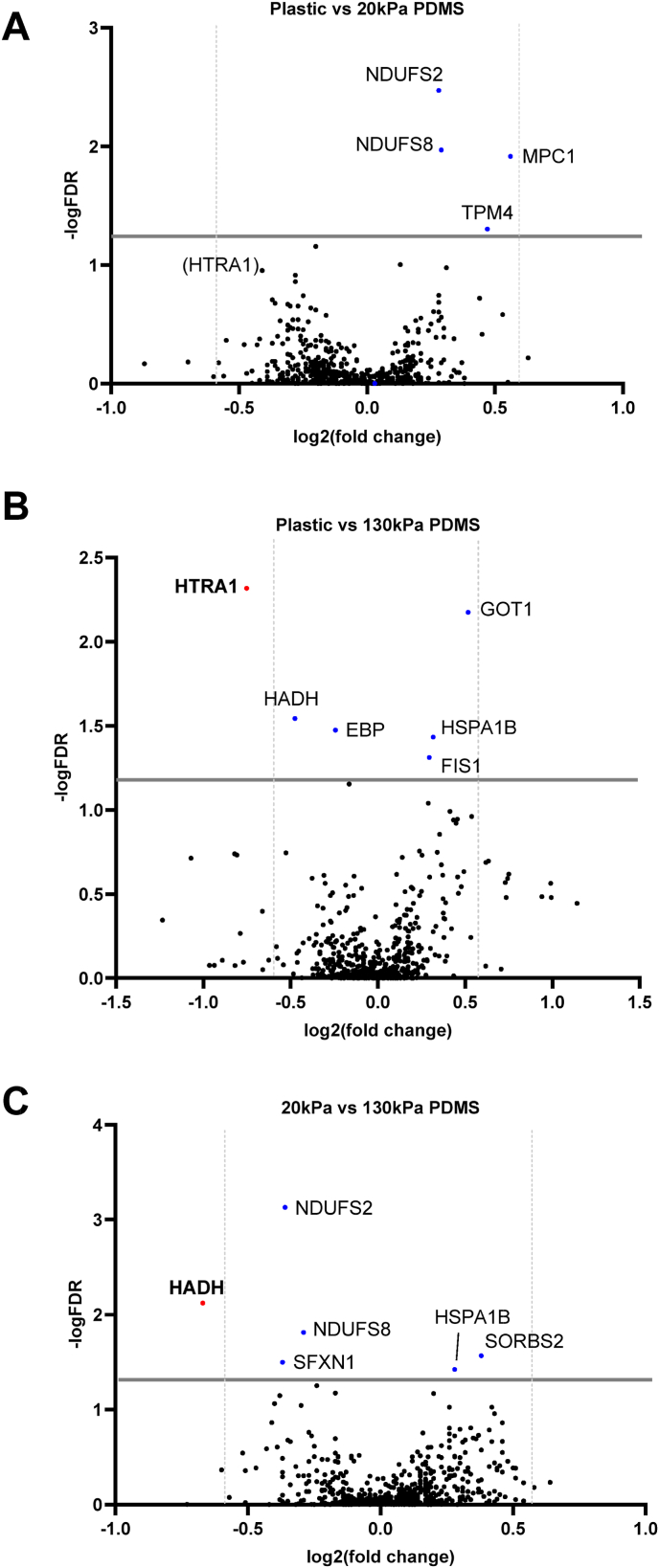
**Proteomics data of differentially up or downregulated proteins of iPSC-CMs cultured on 20 kPa, 130 kPa PDMS and plastic.** Proteomics plots of (A) Plastic vs 20 kPa PDMS (B) Plastic vs 130 kPa PDMS (C) 20 kPa PDMS vs 130 kPa PDMS. On the x axis, 0 indicates no change in protein expression, dots to the right show upregulation of proteins, dots to the left indicate downregulation of proteins. The solid grey line at 1.3 FDR refers to a significance threshold, adjusted p value of 0.05. The dashed grey line refers to log fold change threshold values of >1.5x control. Blue dots refer to proteins that have a p value of <0.05, but do not have a fold change of greater than 1.5x. Red dots refer to proteins that are significantly differentially expressed compared to control (p < 0.05) and greater than 1.5x fold change. Data was obtained from 1 well of cells per condition across n = 4 batches of iPSC-CM differentiations. For protein names see Supplementary Table S3.

Comparisons between iPSC-CMs cultured on plastic and 20 kPa PDMS revealed four proteins of interest, all of which were significantly different according to adjusted p values (<0.05) but did not meet the criteria for log fold change threshold (Supplementary Table 3). Of these, two proteins (NDUFS2 and NDUFS8) were involved in the ETC (Fig. 5A), acting as NADH dehydrogenase proteins which play a role as part of complex I of the ETC.

Protein characterisation of iPSC-CMs cultured on plastic and 130 kPa PDMS highlighted six proteins of interest, with one protein displaying differential expression at both fold change and statistical thresholds (Fig. 5B). Serine protease (HTRA1) was identified as significantly downregulated in iPSC-CMs on plastic, with an adjusted p value of 0.005 and ∼two-fold change (Supplementary Table 3).

When comparing between both 20 kPa and 130 kPa PDMS substrates, six proteins of interest were identified in iPSC-CMs, with hydroxyacyl-coenzyme A dehydrogenase (HADH) differentially downregulated with an adjusted p value of 0.0075 and a ∼two-fold (Fig. 5C–Supplementary Table 3). The other five proteins reached significance for adjusted p values, but did not meet significant fold change criteria.

### 3.5 Transcript expression of metabolic genes

To assess transcriptional changes in metabolic genes, key genes were assessed by qPCR.

**Fig. 6:**
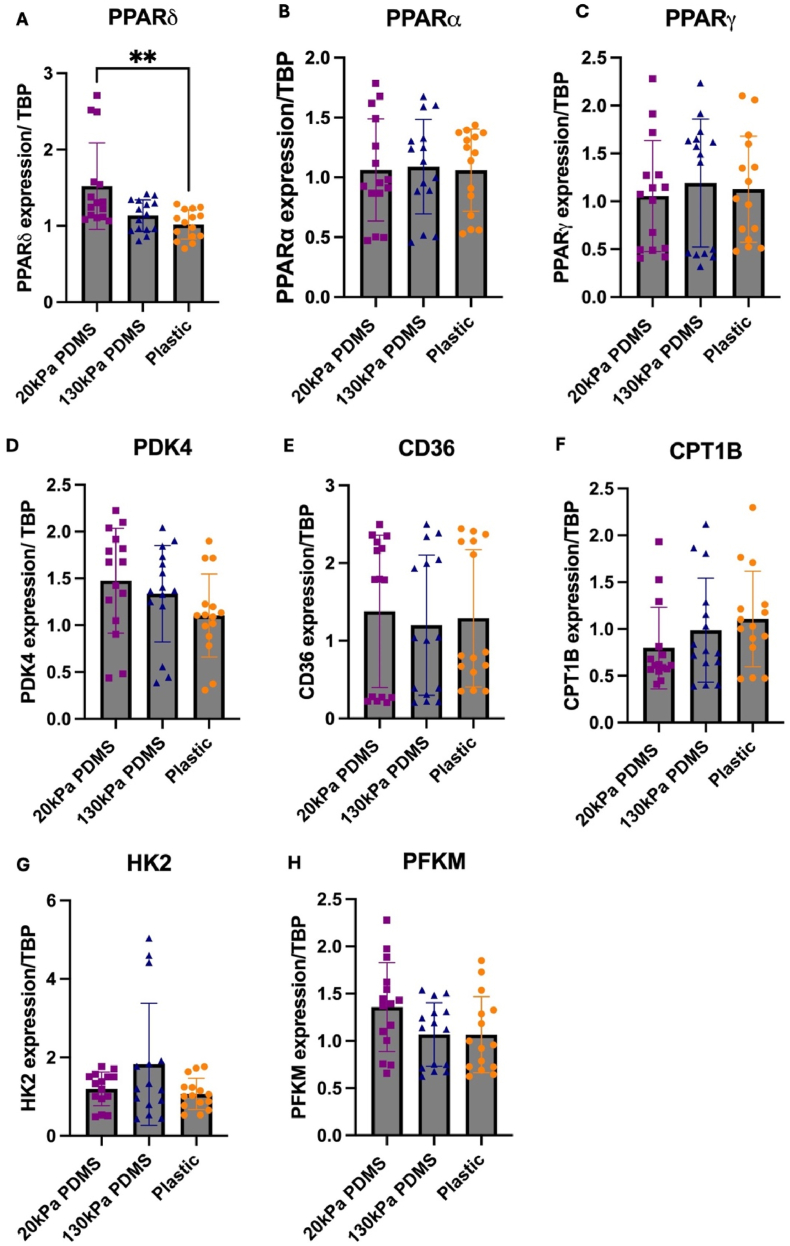
**qPCR transcript expression of metabolic genes in day 25 iPSC-CMs cultured on 20 kPa, 130 kPa PDMS and plastic.** Transcript levels of (A) *PPARδ* (B) *PPARα* (C) *PPARγ* (D) *PDK4* (E) *CD36* (F) *CPT1B* (G) *HK2* (H) *PFKM*. Data has been normalised to *TBP* and made relative to plastic. Values are presented as mean ± SD. Each data point refers to 1 well of cells plated as 3 technical replicates, with a total of n = 5 batches of differentiated iPSC-CMs per condition. Statistical significance was assessed with a Kruskal-Wallis test as data was not normally distributed, with p values determined as <0.01 (∗∗).

A significant increase in *PPARδ* transcript expression was observed in iPSC-CMs cultured on 20 kPa PDMS compared to plastic (p < 0.01) (Fig. 6A). No significant differences in *PPARα* or *PPARγ* transcript expression levels were found in iPSC-CMs cultured on different substrates (Fig. 6 B&C). *PDK4*, responsible for inhibiting the pyruvate dehydrogenase complex and enhancing fatty acid oxidation displayed no significant differences in expression in iPSC-CMs on 20 kPa PDMS compared to plastic (Fig. 6D). Transcript levels of *CD36*, a fatty acid transporter, showed no significant changes across conditions (Fig. 6E). *CPT1B* coding for carnitine palmitoyltransferase 1B is responsible for the transport of long chain fatty acyl-CoAs for the β-oxidation pathway. It showed no significant differences in iPSC-CMs across conditions (Fig. 6F). No significant changes in *HK2* or *PFKM* were seen in iPSC-CMs cultured on substrates (Fig. 6 G&H).

### 3.6 Protein expression of metabolic genes

**Fig. 7:**
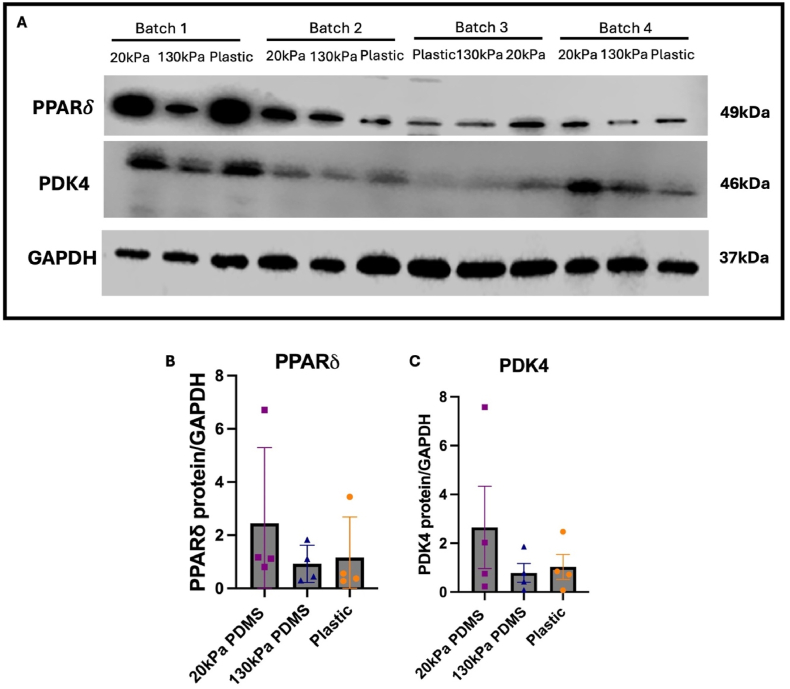
**Protein expression of metabolic genes.** (A) Western blot of PPARδ, PDK4 and GAPDH (B) Quantification of PPARδ expression. (C) Quantification of PDK4 expression. Data has been normalised to GAPDH and made relative to plastic. Values are presented as mean ± SD. Each data point refers to 1 well of cells per condition, across a total of n = 4 batches of iPSC-CM differentiations. Statistical significance was assessed with a Kruskal-Wallis test as data was not normally distributed, with no significant differences observed.

To confirm qPCRs findings at protein level, western blotting was performed. Consistent with transcript levels, PPARδ exhibited an increased trend of expression on 20 kPa PDMS compared to other substrates, although not significant (Fig. 7 A&B). PDK4 displayed the same trend as seen at transcript level, with a trend towards increased expression in iPSC-CMs cultured on 20 kPa PDMS compared to 130 kPa PDMS and plastic, although not significantly different (Fig. 7C).

## 4 Discussion

This paper aimed to investigate the metabolic remodelling associated with substrate stiffness of the ECM on iPSC-CMs, as substrate stiffness is relevant for cardiac pathologies. A key characterisation of the effect of substrate stiffness on iPSC-CMs was the significant shift in metabolic source to meet energetic demand. In failing hearts, a compensatory response to a lack of mitochondrial oxidation and energy deficit is an upregulation of glycolysis (Lopaschuk et al., 2010). This was reflected in iPSC-CMs cultured on stiff plastic substrates compared to softer PDMS substrates, which exhibited greater glycolytic metabolism and anaerobic respiration with upregulated lactate and pyruvate production both at an intracellular and extracellular level. Substrate stiffnesses appear to significantly alter energetics of iPSC-CMs particularly when cultured on surfaces of physiological stiffnesses, highlighting how ECM stiffness can contribute to cardiac disease such as heart failure.

### 4.1 Glycolytic preference of iPSC-CMs cultured on stiff substrates

iPSC-CMs cultured on stiffer substrates demonstrated a shift towards glycolytic metabolism, reflective of cardiac pathologies when assessed through intracellular metabolites. Significantly higher levels of U-^13^C-glucose utilised for pyruvate and lactate production were identified in iPSC-CMs cultured on plastics compared to softer substrates, indicating greater levels of glycolysis. These findings are consistent with literature, showing iPSC-CMs cultured in high glucose media labelled with U-^13^C-glucose have high lactate production and a high fraction of labelled glucose in lactate (Correia et al., 2017). This was further reflected in the data, as despite supplementation of media with OA and PA, iPSC-CMs demonstrated a preference for glycolysis on stiffer substrates. Additionally, total intracellular metabolite levels of lactate and pyruvate in iPSC-CMs cultured on plastic compared to PDMS substrates were higher, suggesting not only alterations in glucose utilisation, but also changes in metabolite utilisation with substrate stiffness. To further characterise changes associated between substrate stiffness and glycolytic lactate production, the activity and expression of lactate dehydrogenase could be investigated.

Consistent with mass spectrometry data, extracellular flux measurements of iPSC-CM bioenergetics on stiff substrates also indicated a higher level of glycolytic activity. The significantly higher basal PER level identified in iPSC-CMs cultured on plastic compared to both softer PDMS substrates reflects higher glycolytic activity. Increased glycolytic index in iPSC-CMs cultured on plastic further reflects this. Furthermore, the increased total ATP production of iPSC-CMs cultured on plastic suggests a higher ATP demand, which is met by increased glycolytic activity. Furthermore, iPSC-CMs on plastic demonstrated a higher glycolytic capacity compared to those cultured on PDMS substrates, as shown by the higher mitochondrial activity after treatment with Monensin, consistent with a glycolytic phenotype.

This is further reinforced by the lack of changes in basal oxygen consumption rate, indicating no changes in mitochondrial respiratory activity across substrates. Increased glycolysis and lactic acid production is typically upregulated in failing, ischemic hearts (Dong et al., 2021; Nyberg and Jones, 2022), reinforcing the disease state of iPSC-CMs on stiffer ECMs.

The disease phenotype of iPSC-CMs on stiff substrates is further reflected by the significantly increased proton leak levels in iPSC-CMs cultured on plastic compared to 20 kPa PDMS. Increased proton leak has been observed in aged mouse mitochondria, with increased proton leak in aged cardiomyocytes occurring through the ANT1 protein (Zhang et al., 2020). In addition, increased proton leak has been identified in cases when the heart is under oxidative stress, such as ischemic reperfusion injury, thus suggesting proton leak is linked with increased reactive oxygen species production and decreased viability of CMs (Murphy and Steenbergen, 2008; Quarrie et al., 2011). The data demonstrates a strong metabolic characterisation of a glycolytic, aged disease phenotype of CMs on stiff substrates, indicating signs of mitochondrial damage and a metabolic substrate switch with increased stiffness.

### 4.2 Protein disease profile of iPSC-CMs cultured on stiffer substrates

Signs of a cardiac disease profile were also demonstrated through proteomics data of iPSC-CMs cultured on plastic. Mitochondrial fission protein 1 (FIS1) upregulation on stiffer substrates indicates a stressed state, as FIS1 is responsible for regulating mitochondrial fission and has been shown to lead to mitochondrial fragmentation and disruption of the myocardial network through cell apoptosis (Li et al., 2020). Upregulation of these proteins suggests iPSC-CMs cultured on stiffer substrates may be undergoing cellular stress, and consequently begin to indicate signs of cardiac dysfunction.

The shift from fatty acid oxidation to glycolysis in heart failure is represented by changes in levels of HADH, which regulates fatty acid utilisation (Wang et al., 2022); the downregulation of HADH in iPSC-CMs cultured on plastic compared to 130 kPa PDMS indicates a reduction in the utilisation of fatty acids and breakdown through β-oxidation. However, HADH was differentially downregulated on 20 kPa PDMS compared to 130 kPa PDMS, contradicting previous evidence of increased fatty acid utilisation on softer substrates. The lack of changes in oxidative phosphorylation across substrate stiffnesses may provide an explanation for this, as this may translate to the lack of significant differences in the amount of β-oxidation occurring.

Downregulation of serine protease HTRA1, an ECM protein involved in collagen 1 secretion and degradation, was identified in iPSC-CMs cultured on plastic compared to 130 kPa PDMS. Increased HTRA1 expression has been identified in patients with dilated cardiomyopathy and is strongly correlated with fibrosis (Shi et al., 2024). The downregulation of HTRA1 may lead to the decreased degradation of ECM proteins, thus consistent with the increased collagen deposition and increased stiffness observed in diseased hearts. Despite the lack of significantly differentially expressed proteins, the identified proteins may reflect the subtle changes that stiffness of the myocardium can have on iPSC-CM proteins, with stiffness potentially altering proteins involved in metabolic stress and cardiac disease. Further investigation into specific proteins and mechanistic pathways may provide insight into stiffness related changes.

### 4.3 Substrate stiffness affects iPSC-CMs metabolic gene expression

In addition to changes in metabolic function and protein expression, substrate stiffnesses also impact transcript expression of genes. The effect of ECM stiffness on PPAR regulation and downstream metabolic pathways are largely undescribed. Much of our understanding of the roles of the PPAR isoforms, PPARα, PPARδ and PPARγ are in metabolic regulation, as they play roles in insulin sensitivity, glucose homeostasis and oxidation of fatty acids and cardiac disease (Lee et al., 2017).

An interesting finding was the significantly increased transcript expression of PPARδ in iPSC-CMs cultured on 20 kPa PDMS compared to plastic. Ligand-mediated activation of PPARδ has been shown to switch energy production to fatty acid oxidation by increasing fatty acid uptake and catabolism (Liu et al., 2018), with previous studies showing increased expression of PPARδ in iPSC-CMs supplemented with fatty acids (Correia et al., 2017; Feyen et al., 2020). Increased expression and activation of PPARδ has been linked to iPSC-CM maturation, with increased myofibrillar alignment, greater contractility, electrophysiological maturity and enhanced metabolic modifications to switch to fatty acid oxidation rather than glycolysis (Wickramasinghe et al., 2022).

Future work will validate increased expression of PPARδ on 20 kPa PDMS at protein and functional level, e.g. by using specific inhibitors (Toth et al., 2012), as increased PPARδ signalling would be consistent with an increased maturation state of iPSC-CMs on softer substrates. Literature has also shown PPARδ activation in iPSC-CMs increases expression of genes linked to fatty acid oxidation, such as PDK4 and CD36 (Wickramasinghe et al., 2022). The increased trend of PDK4 at protein and gene expression level in iPSC-CMs cultured on 20 kPa PDMS in our study also hints at a potential role of increased PPARδ signalling.

The stiffness linked shift in metabolism of iPSC-CMs from fatty acid oxidation to glycolytic activity may occur through several pathways and mechanisms. Potential mechanisms of substrate stiffnesses regulating glycolysis involve rho/ROCK actin pathway (Ge et al., 2021), alterations in phosphofructokinase (Park et al., 2020) or translocation of the GLUT4 transporter (Ge et al., 2021). In addition, increased ECM stiffness has been shown to activate YAP/TAZ signalling, which promotes glycolysis in CMs and regulates glycolysis through several mechanisms (Kashihara and Sadoshima, 2024). It is possible that these pathways could be altered in iPSC-CMs on substrates, thus shifting metabolic activity. Further research is required to determine mechanistic pathways of metabolic activity altered by stiffness of the ECM.

### 4.4 Maturation of iPSC-CMs on softer substrates

Cardiac maturity of iPSC-CMs on softer 20 kPa PDMS gels, representative of a healthy ECM displayed greater cardiac maturity, with increased *MYL2/MYL7* ratios compared to stiffer plastic conditions, confirming isoform switching from immature to adult CM phenotypes (Murphy et al., 2019). This finding is consistent with literature showing physiological PDMS stiffnesses can induce maturation of iPSC-CMs (Dhahri et al., 2022).

Structural organisation of sarcomeres, which are the contractile unit of CMs, are indicative of cardiac maturity. iPSC-CMs cultured on soft 20 kPa substrates had greater sarcomere organisation and alignment, indicating greater maturity. In support, iPSC-CMs on soft 20kPA substrates had a tendency to generate more force (measured as ‘Contraction Amplitude’ Supplementary Figure 5 A) (Guo and Pu, 2020; Hersch et al., 2013). Moreover, they had a trend towards slower beating rates (Supplementary Figure 5 C), indicative of improved maturation (Wang et al., 2021).

The clear disorganisation and lack of α-actinin arrangement in iPSC-CMs cultured on stiffer substrates corresponds with the lack of maturity observed at transcript level, further supports the use of softer substrates as a maturation strategy for iPSC-CMs. This is consistent with other studies which have shown substrate stiffness can affect the structural enhancement of iPSC-CMs (Jimenez-Vazquez et al., 2022; Rao et al., 2013). The combined benefit of softer substrates for physiological relevance as well as iPSC-CM maturation is therefore of importance.

A study using substrate stiffness modulation based on fine-tuned polyacrylamide gels (ranging from ∼4 to 100 kPa), identified ∼50 kPa as the optimal stiffness for cell size, while contractility stress increased with stiffness (Hazeltine et al., 2012). The different chemistry and iPSC-CM lines used and lack of metabolic investigations in the study make a side-by-side comparison of results regarding optimal stiffness difficult.

Furthermore, it is worth noting that previous studies showed beneficial effects of substrate modulation on substrates with higher stiffness than used in this study, e.g. PDMS of 400 kPa stiffness improved maturation in large scale productions compared to plastic (Dhahri et al., 2022), while PDMS films of 1000 kPa stiffness improved electrophysiological characteristics of iPSC-CMs compared to glass (Herron et al., 2016). Both studies highlight the fact that standard culture conditions of iPSC-CMs on plastic or glass with >100 MPa stiffness are highly artificial systems, which may confound experimental outcomes.

### 4.5 Limitations

There are several limitations that need to be considered when evaluating this data.

Immaturity of iPSC-CMs is an intrinsic, well established issue (Krogulec et al., 2025) and replication of key findings using adult cardiomyocytes would add confidence to the data. However, the use of adult rodent cardiomyocytes comes with its own limitations (Krogulec et al., 2025; Saleem et al., 2020) and there is a scarcity of healthy human cardiac tissue to isolate viable cells for long term experiments.

Moreover, the culture of iPSC-CMs in 11 mM glucose levels are considerably higher than physiological glucose levels. The high glucose may therefore be skewing the iPSC-CMs towards a glycolytic phenotype, although glycolytic phenotypes remained even with fatty acid supplementation. Future experiments could be conducted with low (5 mM) glucose levels and fatty acid supplementation to represent physiological levels and show more distinct changes.

The Seahorse assay displayed huge variability between replicates, with some wells displaying expected metabolic profiles after drug injections, whereas others showing different profiles. The physical thickness of the PDMS layer interfered in those cases. To overcome this issue, wells that were clearly observed as not effectively injected with inhibitors were excluded from analysis, but in future, being able to generate thinner, consistent layers of PDMS would be the aim.

Proteomics data quality can vary largely due to variation in sample preparation (Garbis et al., 2005), pH conditions and labelling efficiency (Hutchinson-Bunch et al., 2021). To mitigate this, all samples were processed and carried out in parallel under the same conditions, reducing the variability in samples. Data analysis and thresholds such as FDR, fold change and p-value significance (p ≤ 0.05) can vary with experiments and are typically set to reduce false positives (The et al., 2016). These limitations were overcome by setting fold change thresholds of 1.5, similar to those seen in previous studies conducting proteomics using iPSC-CMs (Cai et al., 2019). In terms of relatively low number of proteins identified in total, there is clear scope for optimisation of the proteomics workflow.

A key limitation of using iPSC-CMs is the variability in the data. The differentiation process of iPSC-CMs consists of several steps, which can alter the quality of differentiations. In order to overcome this, batches of iPSC-CMs were differentiated in parallel with high quality control thresholds in place and biological and technical replicates used for experiments.

### 4.6 Future work

The current study has not addressed how iPSC-CMs sense substrate stiffness at the ECM. Integrins provide the direct connection of cardiomyocytes to ECM molecules. Integrin beta1 signalling has been shown to mediate stiffness associated maturation of iPSC-CMs (Herron et al., 2016; Li et al., 2024). Downstream, talin is a key integrin-associated, stiffness mechano-sensor that would warrant investigation (Chen et al., 2019). Moreover, YAP/TAZ signalling has been implicated as a crucial mechano-signalling pathway in numerous tissues, including the heart (Dupont et al., 2011; Limyati et al., 2025). The roles of both pathways should be explored in future studies.

Moreover, initial findings from the proteomics experiments should be followed up with targeted approaches, such as Western blotting and interrogation of signalling pathways.

Future studies elucidating the role of PPARδ in ECM stiffness would be of interest, by utilising PPARδ agonists and antagonists to characterise specific mechanisms or pathways that are altered. The shift in metabolic source with stiffness could be confirmed with iPSC-CMs cultured in low glucose media levels supplemented with fatty acids, which may provide data of further physiological relevance. Changes at protein level may be investigated further, by assessing phosphorylation states of metabolic proteins such as the pyruvate dehydrogenase complex, providing further insight into pathways altered.

## 5 Conclusions

ECM stiffness due to fibrosis and ECM protein deposition can impact behaviour and function of CMs. This study aimed to develop a representative model of cardiac disease by recapitulating stiffness of healthy and fibrotic ECMs using PDMS viscoelastic polymers and comparing conditions to traditional stiff plastics. This study revealed culture of iPSC-CMs on traditional cell culture plastics or glass coverslips displaying pathological metabolism, highlighting the use of physiological substrates for metabolic investigation. ECM stiffness can impact the metabolic function of iPSC-CMs, with iPSC-CMs cultured on stiffer substrates inducing a switch to increased glycolytic metabolism and lactic acid production, reflective of a cardiac disease phenotype at both intracellular and extracellular levels. Furthermore, substrate stiffness can influence the expression of metabolic genes, with suggestion of increased PPARδ transcript expression in iPSC-CMs cultured on softer substrates, indicating a shift towards metabolic maturity. In addition, 20 kPa and 130 kPa PDMS can serve as physiological models for healthy and fibrotic stiffnesses of the ECM, with both stiffnesses demonstrating changes in metabolism and maturity of iPSC-CMs. Further characterisation of metabolic genes such as PPARδ identified from the study at protein expression level and post translational modifications linked to activity, such as phosphorylation status in iPSC-CMs on substrate stiffnesses would be informative. Localisation of key metabolic proteins where relevant and investigation into the activity of these key metabolic enzymes can help confirm metabolic mechanisms and pathways that are altered with substrate stiffness.

## CRediT authorship contribution statement

**Leena Patel:** Writing – review & editing, Writing – original draft, Visualization, Validation, Software, Resources, Project administration, Methodology, Investigation, Formal analysis, Data curation, Conceptualization. **Bryan P. Marzullo:** Writing – review & editing, Software, Resources, Methodology, Formal analysis, Data curation. **Jonathan Barlow:** Writing – review & editing, Software, Resources, Methodology, Formal analysis, Data curation. **Himani Rana:** Resources, Methodology. **Amar J. Azad:** Writing – review & editing, Resources, Methodology, Conceptualization. **Patricia Thomas:** Resources, Methodology. **Daniel A. Tennant:** Writing – review & editing, Supervision, Resources, Methodology, Funding acquisition, Conceptualization. **Katja Gehmlich:** Writing – review & editing, Supervision, Software, Resources, Conceptualization.

## Funding

Leena Patel is recipient of a studentship BB/T00746X/1 from the UKRI Biotechnology and Biological Sciences Research Council (BBSRC) and University of Birmingham funded Midlands Integrative Biosciences Training Partnership (MIBTP). Work in KG's laboratory is supported by a National Centre for the 3Rs/British Heart Foundation (BHF) grant (NC/T001747/1), by the UKRI Medical Research Council (MR/V009540/1) and the BHF (IA/F/23/275037). The Department of Cardiovascular Sciences, University of Birmingham, received an Accelerator Award by the British Heart Foundation (AA/18/2/34218).

## Declaration of competing interest

The authors declare that they have no known competing financial interests or personal relationships that could have appeared to influence the work reported in this paper.

## Data Availability

Data will be made available on request.
