# Supplementary material for: Substrate stiffness-dependent metabolic reprogramming of iPSC-derived cardiomyocytes on physiological PDMS polymers
Source: Metab Eng Commun. 2025 Jul 8;21:e00266. doi: 10.1016/j.mec.2025.e00266 (PMC12281380; doi:10.1016/j.mec.2025.e00266)
Supplement: Multimedia component 1 [file mmc1.pdf]

Substrate Stiffness-Dependent Metabolic Reprogramming of iPSC-Derived Cardiomyocytes on Physiological PDMS Polymers

Leena Patel, Bryan P. Marzullo, Jonathan Barlow, Himani Rana, Amar J.S. Azad, Patricia Thomas, Daniel A. Tennant, Katja Gehmlich

Supplementary Material

Supplementary Table 1. TaqMan probes used for qPCR

TaqMan probes	
**Target gene**	**Assay ID**
**CD36 (FAM-MGB)**	Hs00354519_m1
**CPT1B (FAM-MGB)**	Hs03046298_s1
**GAPDH (VIC-MGB)**	4626317E
**HK2 (FAM-MGB)**	Hs00606086_m1
**PFKM (FAM-MGB)**	Hs01075411_m1
**PDK4 (FAM-MGB)**	Hs01037712_m1
**PPARα (FAM-MGB)**	Hs00947536_m1
**PPARδ (FAM-MGB)**	Hs04187066_g1
**PPARγ (FAM-MGB)**	Hs01115513_m1
**MYH7 (FAM-MGB)**	Hs01110632_m1
**MYH6 (FAM-MGB)**	Hs01101425_m1
**MYL2 (FAM-MGB)**	Hs00166405_m1
**MYL7 (FAM-MGB)**	Hs01085598_g1
**TBP (VIC-MGB)**	Hs00427620_m1

Supplementary Table 2. Cohen’s d effect sizes for parameters with significant changes in Figure 4.

It was calculated based on following formula: Cohen's $d = (M_2 - M_1) / SD_{pooled}$ with $SD_{pooled} = \sqrt{(SD_1^2 + SD_2^2) / 2}$

**(panel) Parameter**	**20 kPa PDMS vs plastic**		**130 kPa PDMS vs plastic**	
	**Cohen’s d**	**p**	**Cohen’s d**	**p**
(D) Basal Proton Efflux Rate	2.707	<0.0001	2.994	<0.0001
(E) Total ATP Synthesis	1.704	<0.0001	1.852	<0.0001
(F) Basal Glycolytic Index	1.879	<0.0001	2.437	<0.0001
(G) Proton Leak	0.143	0.0468	(1.086)	n.s. (0.0610)

Supplementary Table 3. Protein hits in proteomics experiment (Figure 5).

Proteins listed have a p value of < 0.05, but do not have a fold change of greater than 1.5x. Proteins in bold refer to proteins that are significantly differentially expressed compared to control (p < 0.05) and greater than 1.5x fold change (FC).

**Acronym**	**Name**	**p value**	**FC**
**Plastic vs 20 kPa PDMS**			
MPC1	Mitochondrial pyruvate carrier 1	0.0121	
NDUFS2	NADH dehydrogenase [ubiquinone] iron-sulfur protein 2	0.0034	
NDUFS8	NADH dehydrogenase [ubiquinone] iron-sulfur protein 8	0.0107	
TPM4	Tropomyosin alpha-4 chain	0.0498	
**Plastic vs 130 kPa PDMS**			
EBP	3-beta-hydroxysteroid-Delta(8),Delta(7)-isomerase	0.0336	
FIS1	Mitochondrial fission 1 protein	0.0488	
GOT1	Aspartate aminotransferase, cytoplasmic	0.0067	
HADH	Hydroxyacyl-coenzyme A dehydrogenase, mitochondrial	0.0286	
HSPA1B	Heat shock 70 kDa protein 1B	0.0369	
**HTRA1**	**Serine protease HTRA1**	**0.0048**	**0.51**
**20 kPa vs 130 kPa PDMS**			
**HADH**	**Hydroxyacyl-coenzyme A dehydrogenase, mitochondrial**	**0.0075**	**0.49**
HSPA1B	Heat shock 70 kDa protein 1B	0.0378	
NDUFS2	NADH dehydrogenase [ubiquinone] iron-sulfur protein 2	0.0007	
NDUFS8	NADH dehydrogenase [ubiquinone] iron-sulfur protein 8	0.0154	
SFXN1	Sideroflexin-1	0.0316	
SORBS2	Sorbin and SH3 domain-containing protein 2	0.0271	

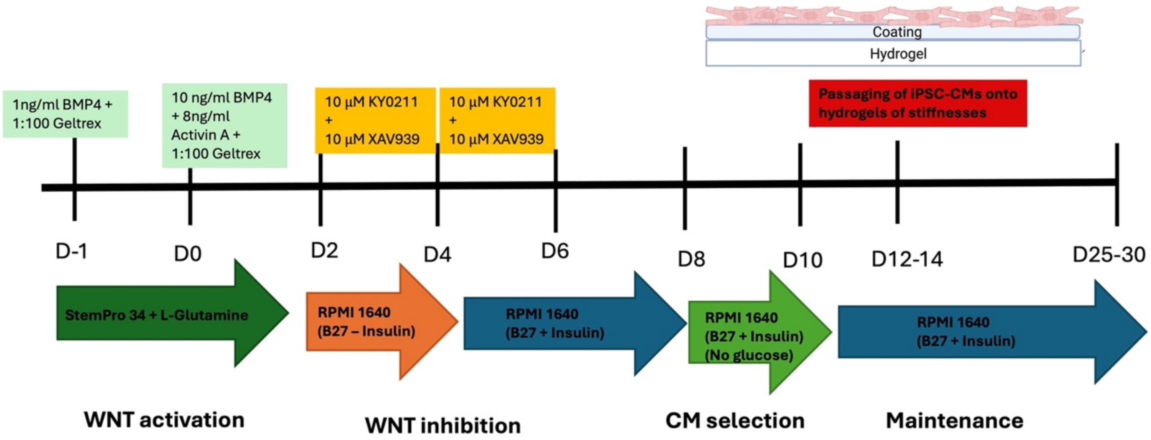

Supplementary Figure 1. Differentiation protocol of iPSCs to iPSC-CMs Schematic diagram of differentiation protocol for iPSC-CMs. The process begins with initiation of day -1 media when iPSCs are approximately 70-80% confluent. Mesoderm differentiation is initiated with BMP4 and Activin A. XAV939 and KY0211 inhibit Wnt signalling, promoting CM development. Selection of CMs occurs during glucose starvation between days 8-10 of differentiation. iPSC-CMs are split onto PDMS viscoelastic polymers on between days 12-14 of differentiation and remain on viscoelastic polymers until day of experiment.

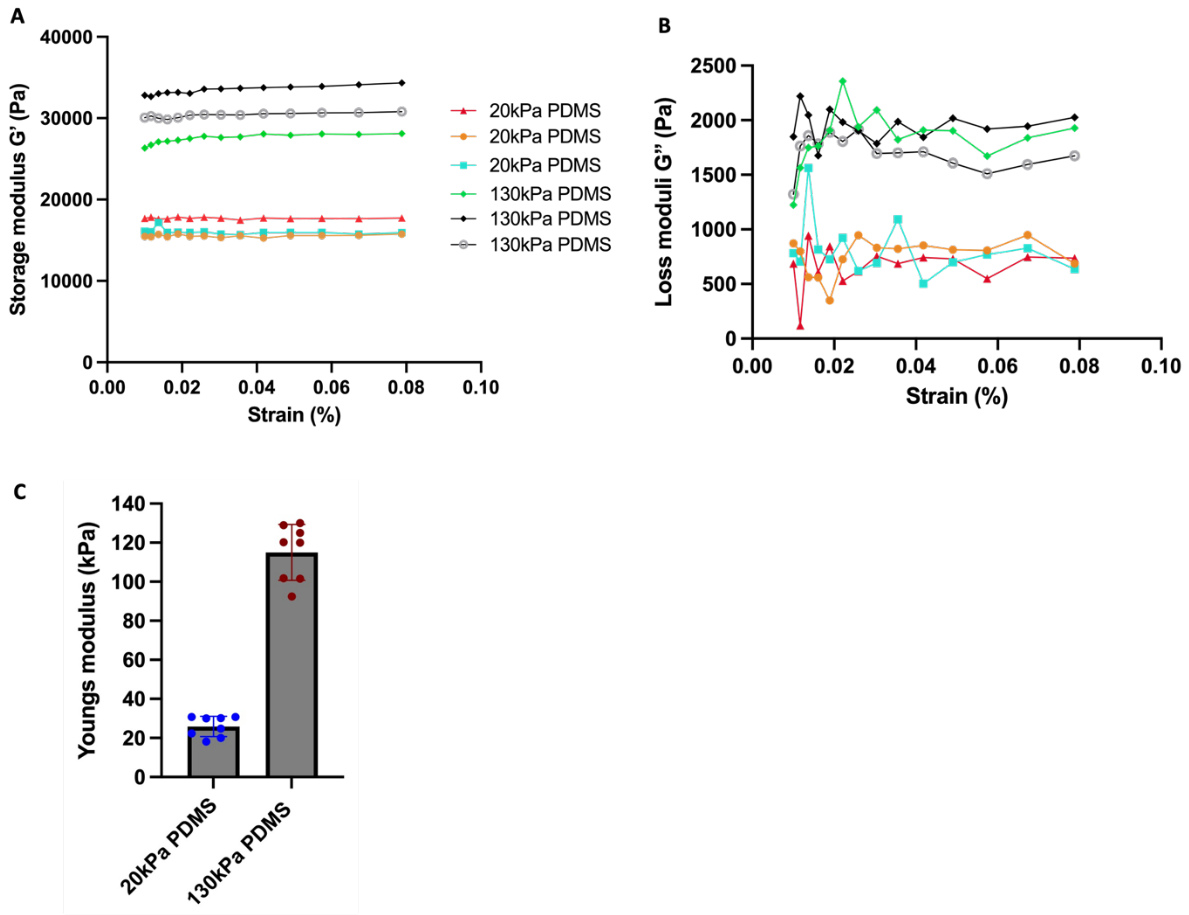

Supplementary Figure 2. **Rheology characterisation of PDMS viscoelastic polymers to confirm stiffnesses**

(A) Storage moduli of 20kPa and 130kPa PDMS viscoelastic polymers (B) Loss moduli of 20kPa and 130kPa PDMS viscoelastic polymers. (C) Young's modulus stiffness of PDMS viscoelastic polymers calculated from storage and loss moduli. Data displayed as mean ± SD, n=3 batches of PDMS viscoelastic polymers. Viscoelastic polymers of each stiffness were measured in duplicate.

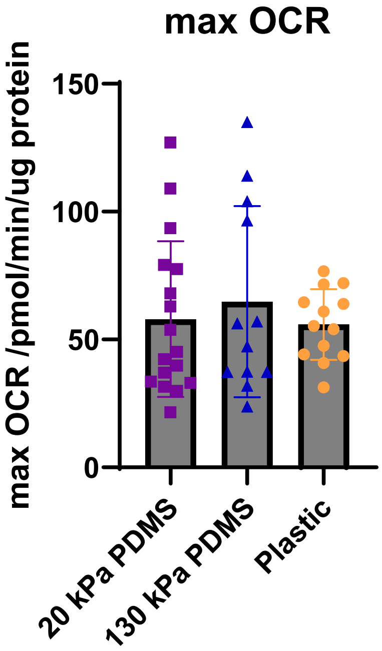

Supplementary Figure 3: Maximum OCR on day 25 of iPSC-CMs cultured on 20kPa PDMS, 130kPa PDMS and Plastic

Quantification of maximum OCR. Values are presented as mean ± SD. Each data point refers to 16 wells of cells per condition per batch, conducted using an n=2 batches of differentiated iPSC-CMs. Statistical significance was assessed with a Kruskal-Wallis test as data was not normally distributed. No significant changes were observed.

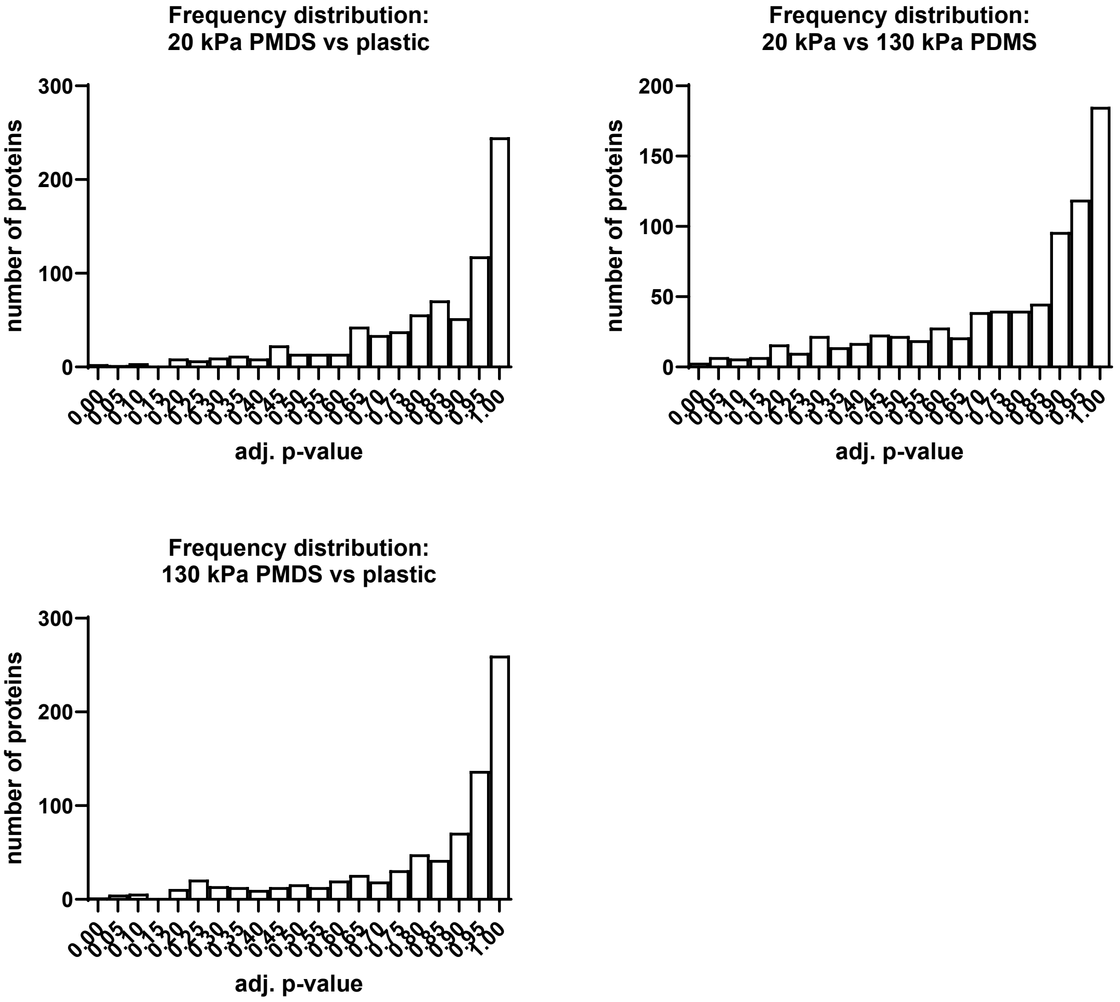

Supplementary Figure 4. Distribution of adjusted p-values of identified proteins in the proteomics experiment for the three comparisons.
Only a small number of identified proteins reaches the threshold of adjusted p-value < 0.05.

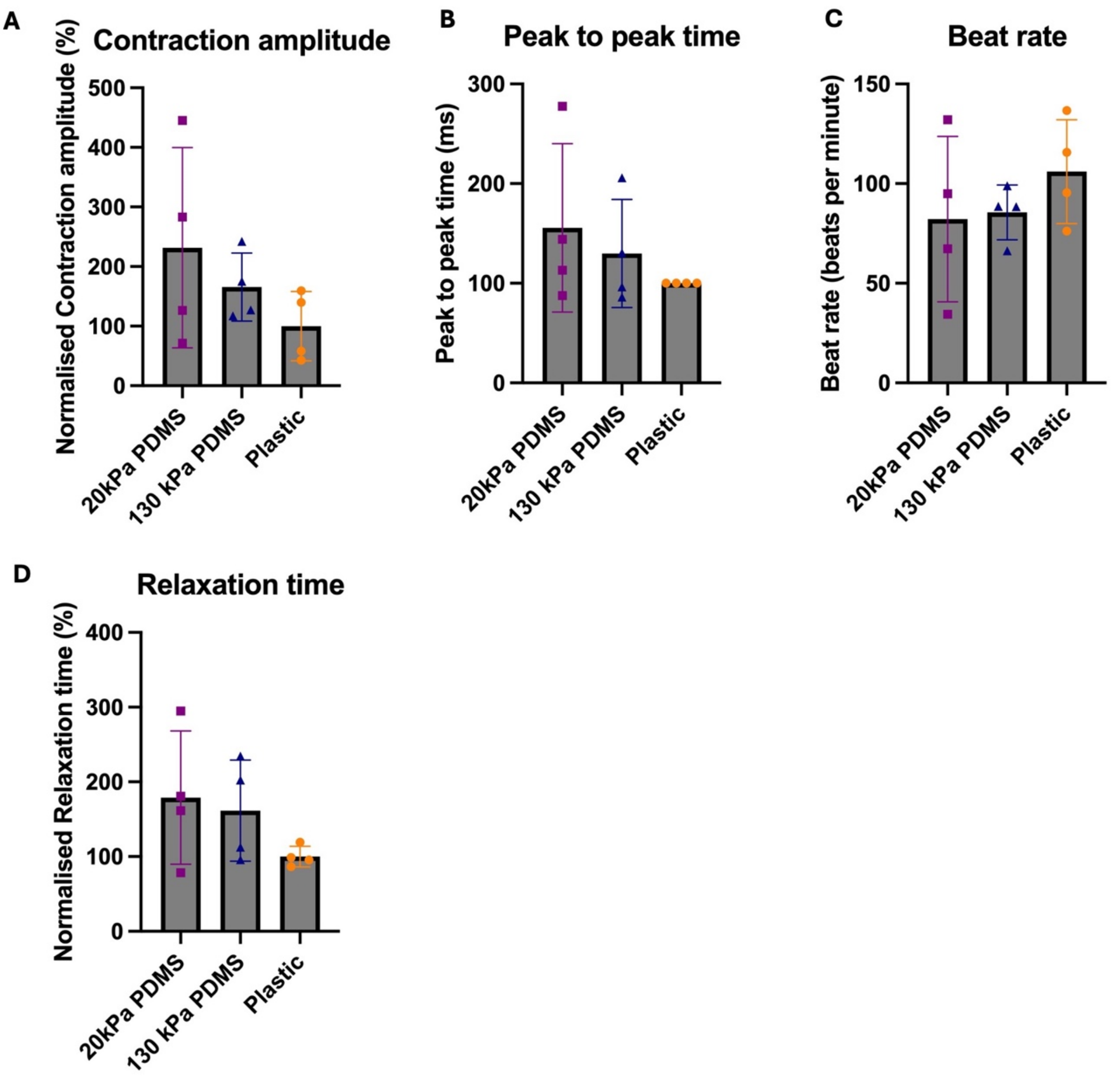

Supplementary Figure 5. Contractility data of iPSC-CMs cultured on 20kPa PDMS, 130kPa PDMS and Plastic assessed by MuscleMotion

(A) Contraction amplitude, referring to the 'force' of contraction (B) Peak to peak time, referring to the time taken between each peak of contraction (C) Beat rate, amount of contractions per minute calculated from contraction duration (D) Relaxation time, referring to the time taken for iPSC-CMs to relax from contraction. Data presented as mean ± SD. Each data point refers to an average of 2-3 wells per condition, with 2-3 different areas recorded per well, across a total of n=4 batches of iPSC-CM differentiations. Average values per condition per batch shown. Data normalised to plastic conditions. Statistical significance was assessed with a Kruskal-Wallis test as data was not normally distributed. No significant differences were observed.
